# Anti-Inflammatory Effect of Ginsenoside Rg5 in Lipopolysaccharide-Stimulated BV2 Microglial Cells

**DOI:** 10.3390/ijms14059820

**Published:** 2013-05-08

**Authors:** Yu Young Lee, Jin-Sun Park, Ji-Sun Jung, Dong-Hyun Kim, Hee-Sun Kim

**Affiliations:** 1 Department of Molecular Medicine and Tissue Injury Defense Research Center, Ewha Womans University Medical School, Seoul 110-783, Korea; E-Mails: lyy1985@ewhain.net (Y.Y.L.); jsp@ewha.ac.kr (J.-S.P.); kurum77@naver.com (J.-S.J.); 2 Department of Life and Nanopharmaceutical Sciences, College of Pharmacy, Kyung Hee University, Seoul 130-701, Korea; E-Mail: dhkim@khu.ac.kr

**Keywords:** ginsenoside Rg5, microglia, neuroinflammation, iNOS, cytokine, signaling pathway

## Abstract

Microglia are resident immune cells in the central nervous system. They play a role in normal brain development and neuronal recovery. However, overactivation of microglia causes neuronal death, which is associated with neurodegenerative diseases, such as Parkinson’s disease and Alzheimer’s disease. Therefore, controlling microglial activation has been suggested as an important target for treatment of neurodegenerative diseases. In the present study, we investigated the anti-inflammatory effect of ginsenoside Rg5 in lipopolysaccharide (LPS)-stimulated BV2 microglial cells and rat primary microglia. The data showed that Rg5 suppressed LPS-induced nitric oxide (NO) production and proinflammatory TNF-α secretion. In addition, Rg5 inhibited the mRNA expressions of iNOS, TNF-α, IL-1β, COX-2 and MMP-9 induced by LPS. Further mechanistic studies revealed that Rg5 inhibited the phophorylations of PI3K/Akt and MAPKs and the DNA binding activities of NF-κB and AP-1, which are upstream molecules controlling inflammatory reactions. Moreover, Rg5 suppressed ROS production with upregulation of hemeoxygenase-1 (HO-1) expression in LPS-stimulated BV2 cells. Overall, microglial inactivation by ginsenoside Rg5 may provide a therapeutic potential for various neuroinflammatory disorders.

## 1. Introduction

Microglia are macrophage-like immune cells in the central nervous system. They are activated in response to brain damage and release various neurotrophic factors to support neuronal cell survival [1]. The activated microglia also release various inflammatory mediators, such as nitric oxide (NO), reactive oxygen species (ROS) and pro-inflammatory cytokines [2,3]. Although microglial activation is essential for host defense in the brain, aberrant activation of microglia can lead to disastrous outcomes, such as neuroinflammation, one of the major causes of neurodegenerative diseases [4,5]. Thus, the regulation of microglial activation may be a valuable therapeutic target for neurodegenerative diseases.

Activation of microglia by lipopolysaccharide (LPS), one of the potent stimulators of macrophages, leads to inflammatory responses by inducing a signal transduction event of the Toll-like receptor 4 (TLR4) [6]. This event leads to the activation of the mitogen-activated protein kinases (MAPKs) pathway and inflammation-associated transcription factors, resulting in induced expression and release of inflammatory mediators, including NO, ROS and pro-inflammatory cytokines, such as tumor necrosis factor-α (TNF-α) and interleukin-1 (IL-1β) [7,8]. In addition, the PI3K/Akt pathway is involved in the activation of NF-κB associated with ROS production in LPS-stimulated BV2 cells [9]. Also, ROS production is closely related to expressions of the antioxidant enzyme HO-1 and NQO1, which are becoming key molecules for regulating oxidative stress and inflammatory responses in the periphery and central nervous systems [10,11]. Previous studies reported that HO-1 and NQO1 are the downstream genes regulated transcriptionally by the Nrf2/ARE (antioxidant response element) pathway [11,12]. Thus, understanding the mechanisms of these pathways is important for controlling inflammatory reactions in activated microglia.

**Figure 1:**
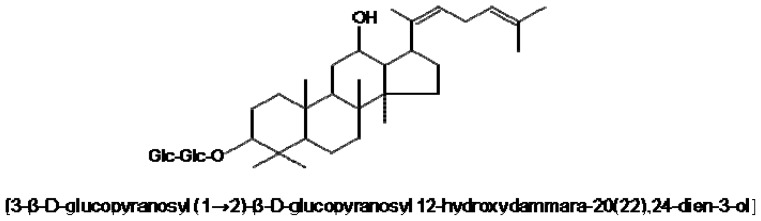
Chemical structure of ginsenoside Rg5.

Ginsenoside Rg5 (Figure 1) is one of the main constituents of steamed ginseng and belongs to protopanaxadiol ginsenosides [13,14]. Rg5 is produced during the steaming and heating processes of the rhizome of *Panax ginseng* CA Meyer, which has been traditionally used in Asian countries for prevention and treatment of various diseases, such as diabetes, cancer, hypertension and allergic diseases [15,16]. Previous studies have reported that Rg5 has anticancer, radical scavenging, anti-dermatitic, platelet anti-aggregating and neuroprotective activities [17–21]. However, the effect of Rg5 on microglial activation has not been reported thus far. Therefore, in the present study, we investigated the anti-inflammatory effects of Rg5 in LPS-stimulated microglia and analyzed the detailed molecular mechanisms.

## 2. Results

### 2.1. Ginsenoside Rg5 Inhibited LPS-Induced NO Production and iNOS Expression in BV2 Microglial Cells

**Figure 2:**
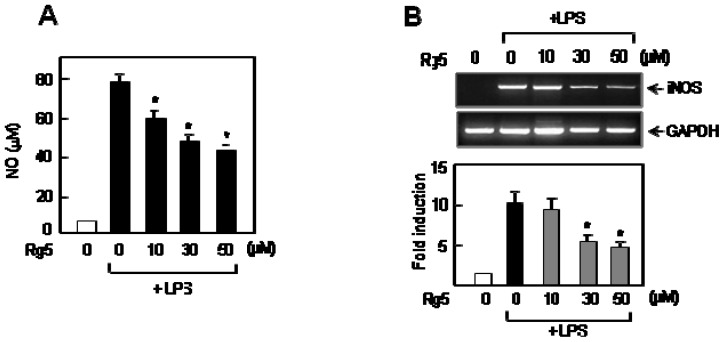
Effect of Rg5 on LPS-induced NO production and iNOS expression in BV2 microglial cells. (**A**) The cells were pre-treated with the indicated concentration of Rg5 for 1 h, followed by treatment of lipopolysaccharide (LPS) (100 ng/mL) for 16 h. The amounts of NO released into media were measured as described in the method section. The data are expressed as the mean ± SEM. of three independent experiments. *p < 0.05, significantly different from the LPS-treated sample; (**B**) RT-PCR data for iNOS mRNA expression. The data are representative of at least three independent experiments. Quantification data are shown in the graph (*n =* 3).

A pro-inflammatory condition of microglia is characterized by excessive release of NO, ROS and pro-inflammatory cytokines [2]. We first examined the effect of Rg5 on NO production in BV2 cells. As shown in Figure 2A, treatment of Rg5 suppressed NO production induced by LPS. Next, we investigated the effect of Rg5 on the mRNA level of iNOS, an enzyme involved in the synthesis of NO in microglia. We also observed the inhibitory effect of Rg5 on LPS-induced mRNA expression of iNOS (Figure 2B). The data suggests that Rg5 regulates iNOS expression at a transcriptional level.

### 2.2. Rg5 Inhibited LPS-Induced Expression of TNF-α and Other Pro-Inflammatory Molecules

**Figure 3:**
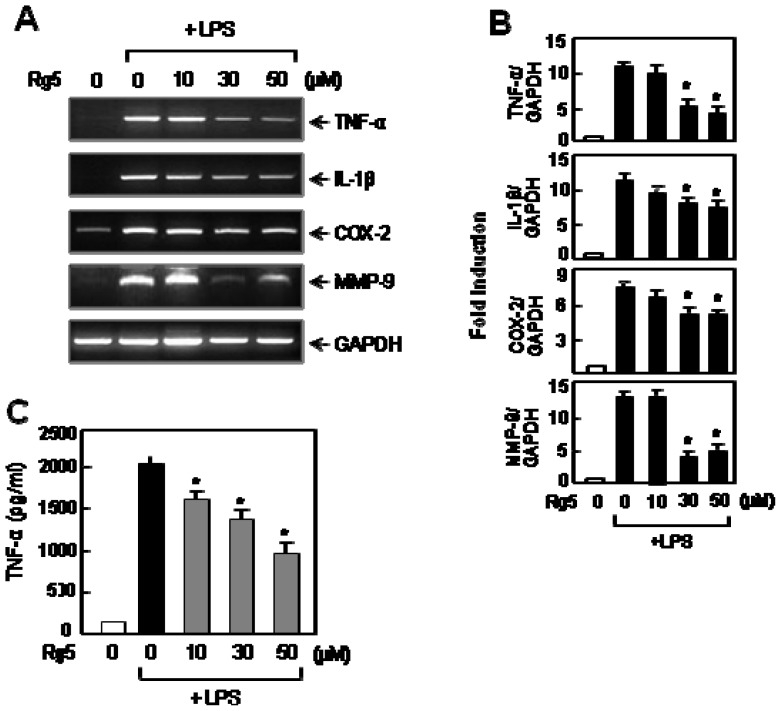
Rg5 suppressed LPS-induced expression of TNF-α, IL-1β, COX-2 and MMP-9 in BV2 cells. (**A**) The cells were pre-treated with the indicated concentration of Rg5 for 1 h, followed by treatment of LPS (100 ng/mL) for 6 h. Total RNA was isolated from the cells, and RT-PCR was performed to measure the mRNA levels of TNF-α, IL-1β, COX-2 and MMP-9. The data are representative of three independent experiments; (**B**) Quantification data are shown in the graph (*n =* 3). *p < 0.05, significantly different from the LPS-treated sample; (**C**) The BV2 cells were pre-treated with Rg5 A for 1 h, followed by treatment of LPS (100 ng/mL) for 16 h. The amounts of TNF-α released into media were measured by ELISA. The data are expressed as the mean ± SEM. of three independent experiments. *p < 0.05, significantly different from the LPS-treated sample.

Next, we investigated the effect of Rg5 on TNF-α, which plays a key role in inflammatory reactions. RT-PCR and ELISA data showed that Rg5 significantly suppressed mRNA expression of TNF-α and subsequently inhibited the TNF-α protein production in LPS-stimulated BV2 microglial cells (Figure 3A–C). Furthermore, Rg5 inhibited the mRNA expression of IL-1β, COX-2 and MMP-9, which are also important pro-inflammatory molecules (Figure 3A). The results demonstrate the anti-inflammatory role of Rg5 in LPS-stimulated microglia.

### 2.3. Rg5 Inhibited LPS-Induced NO and TNF-α Production in Rat Primary Microglia

**Figure 4:**
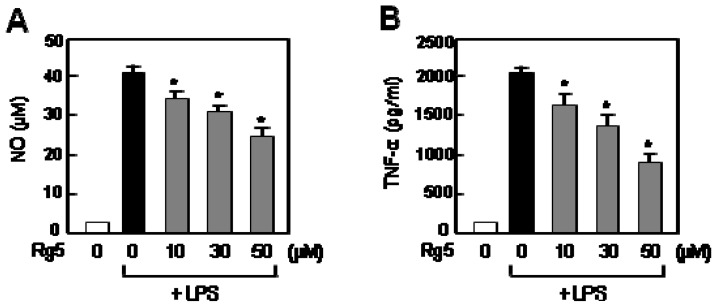
Effect of Rg5 on NO and TNF-α production in LPS-stimulated primary microglial cells. (**A**) The cells were pre-treated with the indicated concentration of Rg5 for 1 h, followed by treatment of LPS (100 ng/mL) for 16 h. The amounts of NO released into media were measured as described in the method section; (**B**) The amounts of TNF-α released into media were measured by ELISA. The data are expressed as the mean ± SEM. of three independent experiments. *p < 0.05, significantly different from the LPS-treated sample.

We further examined the effects of Rg5 on LPS-induced NO and TNF-α production in rat primary microglia. We found that Rg5 significantly inhibited NO and TNF-α production, which is consistent with the results from BV2 cell lines (Figure 4).

### 2.4. The Effects of Rg5 on NO and TNF Production Are Dependent on Pre-Treatment Time of Rg5

**Figure 5:**
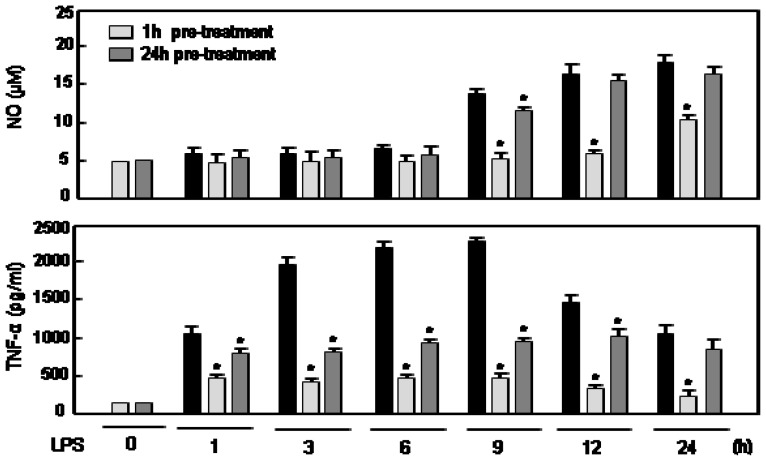
Comparison of the effects of 1 h or 24 h pre-treatment of Rg5 on NO and TNF-α production in LPS-stimulated BV2 microglial cells.

In the above data (Figures 2 and 4), we presented the effects of one hour treatment of Rg5 followed by LPS treatment. To investigate whether pre-treatment of 24 h before the LPS challenge affects the anti-inflammatory effects of Rg5, we compared the effects of 1 h and 24 h pre-treatment of Rg5 on LPS-induced NO and TNF-α production in BV2 cells at various time points. As shown in Figure 5, the 24 h-pre-treated samples showed less anti-inflammatory effects compared with the 1 h-pre-treated group. The attenuation of anti-inflammatory effects of Rg5 in the 24 h pre-treatment group may be explained in the following points of view. First, the target of Rg5 is upstream in the inflammation signaling (e.g., cell surface receptor, NADPH oxidase, *etc.*). Thus, the effect of Rg5 may be reduced after 24 h, because most of the Rg5 is uptaken by the cells at that time point. Second, Rg5 is known to have binding affinity with BSA in the serum of the media, which may cause reduction in the concentration of free Rg5 that acts on the cells. Furthermore, when we examined the time course effect of Rg5, the ginsenoside significantly inhibited TNF-α and NO production at 1 h and 9 h of LPS treatment, respectively, and the effects persisted at least for 24 h (Figure 5). The data indicate that Rg5 modulates upstream inflammatory signaling and, thus, produces anti-inflammatory effects at early time points.

### 2.5. Rg5 Suppressed LPS-Induced DNA Binding Activities of NF-κB and AP-1

**Figure 6:**
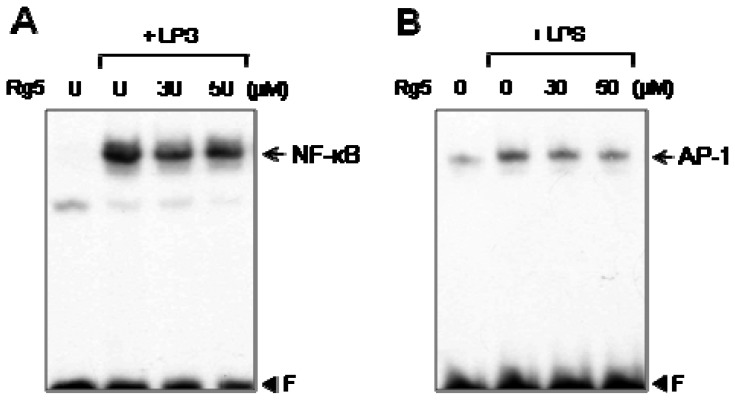
Effect of Rg5 on DNA binding activities of NF-κB (**A**) and AP-1 (**B**) in LPS-stimulated BV2 cells. Nuclear extracts were prepared from BV2 cells after treatment with LPS (100 ng/mL) for 3 h in the absence or presence of Rg5, and then DNA binding activities were examined by electrophoretic mobility shift assay (EMSA). The data show that Rg5 significantly inhibited LPS-induced DNA binding activities of NF-κB and AP-1. The results are representative of three independent experiments.

To further investigate the molecular mechanism underlying the anti-inflammatory effects of Rg5, we examined the effect on NF-κB and AP-1, which are key transcription factors modulating the gene expressions of proinflammatory cytokines and iNOS in microglia [22]. As shown in Figure 6, LPS treatment significantly increased NF-κB and AP-1 DNA binding activities, and Rg5 inhibited both the NF-κB and AP-1 DNA binding. Therefore, the inhibitory effects of Rg5 on NF-κB and AP-1 may be one of the factors contributing to the anti-inflammatory effect of Rg5 in LPS-stimulated BV2 microglia.

### 2.6. Rg5 Inhibited LPS-Induced Phosphorylation of MAPKs and Akt in BV2 Cells

**Figure 7:**
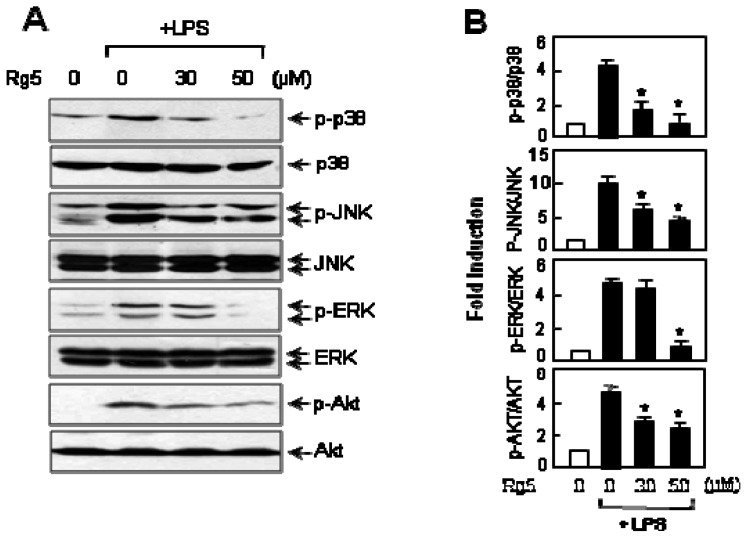
Rg5 inhibits the phosphorylation of three types of MAP kinases and Akt in LPS-stimulated BV2 cells. (**A**) Cell extracts were prepared from BV2 cells treated with LPS (100 ng/mL) for 3 h with or without Rg5 and were subjected to immunoblot analysis using antibodies against phospho- or a total form of three types of MAP kinases (p38 MAPK, ERK1/2 and JNK) and Akt. The results are representative of three independent experiments; (**B**) Quantitative analysis of the phosphorylations of three MAPKs and Akt. The data are expressed as the mean ± SEM. of three independent experiments. *p < 0.05, significantly different from the LPS-treated sample.

MAPKs and PI3K/Akt play the role as upstream signaling molecules in inflammatory reactions [6,9,23]. To investigate whether these signaling pathways are involved in the regulation of microglial activation by Rg5, we examined the effect of Rg5 on the phosphorylation of three types of MAPKs and Akt, a downstream substrate of PI3K. As shown in Figure 7, Rg5 significantly suppressed LPS-induced phosphorylation of ERK, JNK and p38 MAPK. In addition, Rg5 suppressed Akt phosphorylation in LPS-stimulated BV2 cells. The results suggest that both the MAPKs and PI3K/Akt signaling pathways are involved in the anti-inflammatory effect of Rg5 in LPS-stimulated microglia.

### 2.7. Rg5 Inhibited ROS Production with Increase of ARE-Mediated of HO-1 Expression

**Figure 8:**
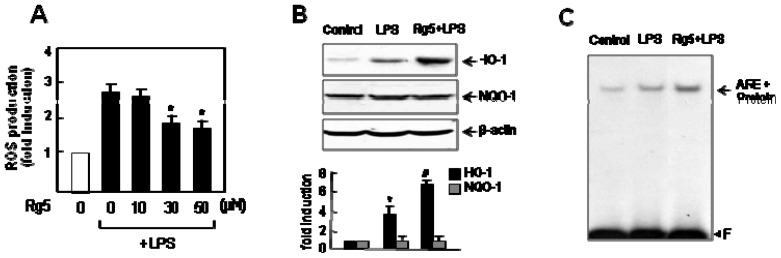
Rg5 inhibited ROS production and increased HO-1 expression via ARE. (**A**) The BV2 cells were pre-treated with Rg5 for 1 h, followed by treatment with LPS (0.1 μg/mL) for 16 h. The intracellular ROS levels were then measured by the DCF-DA method, as described in the Materials and Methods section. The data are expressed as the mean ± SEM. of three independent experiments. *p < 0.05, significantly different from LPS-treated sample; (**B**) Western blot analysis shows the effect of Rg5 (50 μM) on HO-1 and NQO1 protein expressions. The BV2 cells were treated with LPS for 24 h in the absence or presence or of Rg5, and cell lysates were obtained. Quantification data are shown in the graph (*n =* 3). The data are expressed as the mean ± SEM. of three independent experiments. *p < 0.05, significantly different from the control sample. ^#^*p* < 0.05, significantly different from the LPS treated sample; (**C**) EMSA for nuclear protein binding to the ARE element. The arrow indicates a DNA-protein complex of ARE. The data are representative of three independent experiments.

ROS plays the role as early signaling inducer in inflammation [24]. Thus, we examined the effect of Rg5 on ROS production in LPS-stimulated BV2 cells. The DCF-DA fluorescence assay showed that Rg5 significantly attenuated LPS-induced ROS production (Figure 8A). Next, we examined the effect of Rg5 on the expressions of HO-1 and NQO1, which are phase II antioxidant enzymes involved in cellular defense against oxidative stress [10,11]. Western blot analysis showed that Rg5 upregulated HO-1 expression, but did not upregulate NQO1 (Figure 8B). We further examined the effect of Rg5 on the nuclear protein binding to ARE, which is a *cis*-regulatory element that modulates HO-1 expression. As shown in the EMSA data in Figure 8C, Rg5 potentiated nuclear protein binding to ARE. The data collectively suggest that Rg5 exerts antioxidant/anti-inflammatory effects, at least in part by upregulating ARE-mediated HO-1 expression.

## 3. Discussion

In the present study, we report the anti-inflammatory effect of ginsenoside Rg5 in activated microglia. Rg5 inhibited iNOS and TNF-α expression at the mRNA and protein level. Rg5 also inhibited the expressions of proinflammatory molecules, such as IL-1β, COX-2 and MMP-9. Detailed mechanistic studies revealed that Rg5 exerts anti-inflammatory effects by modulating MAPK and PI3K/Akt signaling pathways and their downstream transcription factors, NF-κB and AP-1. Furthermore, Rg5 decreased the intracellular ROS level, which is associated with the upregulation of HO-1 expression.

Previous studies demonstrated that MAPKs play an important role in the LPS-induced expressions of iNOS, COX-2 and pro-inflammatory cytokines in microglia [7,8]. Moreover, PI3K/Akt is reported to be responsible for the LPS-induced production of TNF-α in microglia [25]. Here, we showed that Rg5 inhibited the phosphorylation of three types of MAPKs and Akt, indicating that these signaling molecules are upstream targets of Rg5.

NF-κB and AP-1 are key transcription factors regulating gene expression of pro-inflammatory molecules, such as iNOS, IL-1β and TNF-α [22,26]. In addition, our previous studies and studies by other groups have reported that NF-κB and AP-1 modulates proinflammatory MMP-3 and MMP-9 expressions in activated microglia and astrocytes [27,28]. Thus, the inhibition of NF-κB and AP-1 also contributes to the strong anti-inflammatory effects of Rg5 in activated microglia.

Rg5 also showed antioxidant effects by decreasing the intracellular ROS level, which appears to be related to HO-1 expression via the Nrf2/ARE pathway. Our group recently reported that HO-1 plays an important role in mediating anti-inflammatory/antioxidant effects in microglia. Thus, knockdown of HO-1 aggravated NO and ROS production in LPS-stimulated microglia [29]. The transcription factor Nrf2 binds to several ARE sites on the HO-1 promoter and upregulates HO-1 expression [10]. In the present study, we showed that Rg5 enhanced HO-1 expression via increasing nuclear protein binding to ARE. Thus, the upregulation of HO-1 is also one of the factors contributing to the anti-inflammatory/antioxidant effect of Rg5.

Due to the structural similarity of ginsenosides with steroid hormones, ginsenosides are usually named as steroidal saponins [30]. Studies have proposed that ginsenosides may produce cellular effects by interacting with multi-receptor systems (*i.e.*, steroid receptors, ion channels) or by penetrating into the cells. For example, ginsenoside Rg1 and compound K bind to glucocorticoid receptors and produce neuroprotective or anti-inflammatory effects [31,32]. Although Rg5 receptors are not identified yet, the mode of action of Rg5 might be similar to other ginsenosides. Further studies are necessary to identify Rg5 receptor in the future.

Several papers have reported the therapeutic effects of ginsenoside Rg5 in periphery and central nervous systems. Rg5 improved inflammatory skin disorders, such as oxazolone-induced chronic dermatitis in mice, by modulating IL-1β and TNF-α production in macrophage cells and IFN-γ from Th cells [18]. Rg5 also suppressed inflammation in mice by blocking the interactions of LPS and TLR4 on macrophages, which are associated with inhibition of NF-κB activation [20]. In addition, the hydroxyl radical scavenging activity and anti-cancer effect of Rg5 against lung adenocarcinoma have been reported [14,19]. Regarding the effects of Rg5 on the brain, it was reported that Rg5, along with ginsenoside Rb1 and Rc, protected the striatal neurons in a cellular model of Huntington’s disease by inhibiting glutamate-induced calcium responses [33]. In addition, Rg5 protected scopolamine-induced memory deficits in mice via inhibition of acetylcholine esterase activity and upregulation of BDNF and p-CREB in the brain [21]. The results indicate the effectiveness of Rg5 in the central nervous system.

Besides Rg5, we have previously reported the anti-inflammatory effects of several ginsenosides in brain microglia. It was demonstrated that protopanaxatriol ginsenoside Rh1 penetrated into the mouse brain and inhibited microglial activation [29]. We also showed that the anti-inflammatory effects of Rh1 partly depend on the PKA pathway and hemeoxygenase-1 expressions. In addition, compound K, one of the major metabolites of ginseng, inhibited iNOS and pro-inflammatory mediators in the BV2 cells and primary microglia [34]. Compound K also exerts neuroprotective effects in the ischemic mouse brain via modulating microglial activation [34]. Thus, the anti-inflammatory effects of these ginsenosides along with Rg5 may increase the therapeutic value of ginseng for various CNS disorders.

## 4. Experimental Section

### 4.1. Reagents

All reagents used for cell culture were purchased from Gibco BRL (Grand Island, NY, USA). LPS (*Escherichia coli* serotype 055:B5) were purchased from Sigma Chemical Co. (St. Louis, MO, USA). Antibodies against the phospho-/total form of JNK, p38 MAPK, ERK or Akt were purchased from Cell Signaling Technology (Beverely, MA, USA). Antibodies against HO-1 and NQO1 were purchased from Santa Cruz Biotechnology (Santa Cruz, CA, USA). All reagents used for RT-PCR and oligonucleotides for electrophoretic mobility shift assay (EMSA) were purchased from Promega (Madison, WI, USA). Ginsenoside Rg 5 (Figure 1) was isolated according to the previously reported method [35].

### 4.2. Microglial Cell Culture

The immortalized murine BV2 microglial cell line [36] was grown and maintained in Dulbecco’s modified Eagle’s medium supplemented with 10% heat-inactivated FBS, streptomycin (10 μg/mL) and penicillin (10 U/mL) at 37 °C. Primary microglial cells were cultured from the cerebral cortices of 1–2-day-old Sprague-Dawley rat pups, as described previously [34]. The purity of microglial cultures was >95%, which was determined by isolectin B4 staining (data not shown).

### 4.3. Measurement of TNF-α, Nitrite and Intracellular ROS Levels

Microglial cells (1 × 10^5^ cells per well in a 24-well plate) were pre-treated with Rg5 for 1 h and stimulated with LPS (0.1 μg/mL). The supernatants of the cultured microglia were collected 16 h after LPS stimulation, and the concentrations of TNF-α were measured by an enzyme-linked immunosorbent assay (ELISA), following the procedure recommended by the supplier (PharMingen, San Diego, CA, USA). Accumulated nitrite was measured in the cell supernatant using the Griess reagent (Promega). The intracellular accumulation of ROS was measured with H_2_DCF-DA (Sigma-Aldrich) by modifying a previously reported method [37].

### 4.4. RT-PCR

BV2 cells (7.5 × 10^5^ cells on a 6 cm dish) were treated with LPS in the presence or absence of Rg5, and total RNA was extracted with TRI reagent (Sigma). For RT-PCR, total RNA (1 μg) was reverse-transcribed in a reaction mixture containing 1 U RNase inhibitor, 500 ng random primers, 3 mM MgCl_2_, 0.5 mM dNTP, 1× RT buffer and 10 U reverse transcriptase (Promega). The synthesized cDNA was used as a template for PCR reaction using GoTaq polymerase (Promega) and primers, as shown in Table 1.

### 4.5. Electrophoretic Mobility Shift Assay (EMSA)

Nuclear extracts from treated microglia were prepared as described in a previous study [26]. The double stranded DNA oligonucleotides containing the NF-κB, AP-1 and ARE consensus sequences (Promega) were end-labeled by [γ-^32^P] ATP. Five micrograms of the nuclear proteins were incubated with a ^32^P-labeled probe on ice for 30 min and resolved on a 5% acrylamide gel, as described in a previous study [38].

### 4.6. Western Blot Analysis

Cell extracts were prepared as described previously [38], and the proteins were separated by 12% SDS-polyacrylamide gel electrophoresis and transferred to a nitrocellulose membrane. After blocking, the membranes were incubated with primary antibodies (1:1000); horseradish peroxidase-conjugated secondary antibodies (1:2000 dilution in TBST; New England Biolabs, Ipswich, MA, USA) were then applied, and the blots were developed using an enhanced chemiluminescence detection kit (Thermo Fisher Scientific, Waltham, MA, USA).

### 4.7. Statistical Analysis

Unless otherwise stated, all experiments were performed with triplicate samples and repeated at least three times. The data are presented as the mean ± SEM, and statistical comparisons between the groups were performed using one-way ANOVA, followed by a Student’s *t*-test. A *p*-value of <0.05 was considered significant.

## 5. Conclusions

The present study reports for the first time the anti-inflammatory effect of ginsenoside Rg5 in LPS-stimulated microglia and related detailed molecular mechanisms. Considering that Rg5 is a major component of steamed ginseng with few side effects and low toxicity in the body, Rg5 may be a promising therapeutic candidate for various neuroinflammatory disorders, such as Alzheimer’s disease and Parkinson’s disease.

## Figures and Tables

**Table 1:** Primers used in PCR experiments.

Gene	Forward Primer (5′→3′)	Reverse Primer (5′ →3′)	Size
iNOS	CAAGAGTTTGACCAGAGGACC	TGGAACCACTCGTACTTGGGA	450 bp
TNF-α	CCTATGTCTCAGCCTCTTCT	CCTGGTATGAGATAGCAAAT	354 bp
IL-1β	GGCAACTGTTCCTGAACTCAACTG	CCATTGAGGTGGAGAGCTTTCAGC	447 bp
COX-2	TTCAAAAGAAGTGCTGGAAAAGGT	GATCATCTCTACCTGAGTGTCTTT	304 bp
MMP-9	GTGATCCCCACTTACTATGGAAAC	GAAGCCATACAGTTTATCCTGGTC	352 bp
GAPDH	ATGTACGTAGCCATCCAGGC	AGGAAGGAAGGCTGGAAGAG	420 bp
